# Long-term biopsy outcomes in prostate cancer patients treated with external beam radiotherapy: a systematic review and meta-analysis

**DOI:** 10.1038/s41391-021-00323-6

**Published:** 2021-02-08

**Authors:** Saurabh Singh, Caroline M. Moore, Shonit Punwani, Anita V. Mitra, Steve Bandula

**Affiliations:** 1 Centre for Medical Imaging, University College London, London, UK; 2 Division of Surgery and Interventional Science, University College London, London, UK; 3 Department of Urology, University College London Hospitals NHS Foundation Trust, London, UK; 4 Cancer Services, University College London Hospitals NHS Foundation Trust, London, UK; 5 Interventional Oncology Service, University College London Hospitals NHS Foundation Trust, London, UK

**Keywords:** Outcomes research, Cancer therapy

## Abstract

**Background:**

Biopsy after external beam radiotherapy (EBRT) for localised prostate cancer (PCa) is an infrequently used but potentially valuable technique to evaluate local recurrence and predict long-term outcomes.

**Methods:**

We performed a meta-analysis of studies until March 2020 where a post-EBRT biopsy was performed on patients with low-to intermediate risk PCa, according to the Preferred Reporting Items for Systematic Review and Meta-analysis (PRISMA) statement. The primary outcome was the aggregate post-EBRT positive biopsy rate (≥2 years after EBRT) and the associated odds ratio (OR) of a positive biopsy on biochemical failure (BCF), distant metastasis-free survival (DMFS) and prostate cancer-specific mortality (PCSM). A sensitivity analysis was performed which examined biopsy rate as a function of post-EBRT biopsy protocol, PCa risk, ADT usage and radiation dose.

**Results:**

A total of 22 studies were included, of which 10 were randomised controlled trials and 12 were cohort studies. Nine out of the 22 studies used dosing regimens consistent with the 2020 NCCN radiotherapy guidelines. The weighted-average positive biopsy rate across all 22 studies was 32% (95%-CI: 25–39%, *n* = 3017). In studies where post-treatment biopsy was part of the study protocol, the rate was 35% (95%-CI: 21–38%, *n* = 2450). In the subgroup of studies that conformed to the 2020 NCCN radiotherapy guidelines, this rate was 22% (95% CI: 19–41%, *n* = 832). Patients with positive biopsy had a 10-fold higher odds of developing BCF (OR of 10.3, 95%-CI: 3.7–28.7, *p* < 0.00001), 3-fold higher odds of developing distant metastasis (OR 3.1, 95%-CI: 2.1–4.7, *p* < 0.00001) and 5-fold higher odds of dying from their PCa (OR 5.1, 95%-CI: 2.6–10, *p* < 0.00001).

**Conclusion:**

A positive biopsy after EBRT is associated with a poor prognosis compared to a negative biopsy. The post-EBRT positive biopsy rate is an important measure which provides additional insight when comparing EBRT to other treatment modalities for PCa.

## Introduction

External beam radiotherapy (EBRT) has been a long-standing, recommended primary treatment for prostate cancer (PCa). With advances in delivery and treatment planning, EBRT has become safer, more precise and more effective [1]. Despite these improvements, traditionally defined low- and intermediate-risk PCa patients treated with modern EBRT can still expect biochemical recurrence at 10 years in 10% and 23% of cases, respectively [2]. In addition to identifying recurrence as early as possible, it is also important to establish its location. PCa that has metastasised has different management options compared with radiorecurrent disease that is still confined to the prostate [3, 4].

Biochemical failure (BCF) after EBRT is the most established predictor of PCa recurrence. BCF is currently assessed using the Phoenix criteria, defined as a rise by 2 ng/ml or more above nadir prostate specific antigen (PSA) levels [5]. Despite its obvious benefits, the primary drawback of BCF is that it does not discriminate between locally recurrent disease and metastasis. Increasingly for the assessment of local tumour control, imaging and biopsy are used [4]. Both multiparametric magnetic resonance imaging (mpMRI) and prostate specific membrane antigen-positron emission tomography (PSMA-PET) have shown promise in detecting residual or recurrent cancer after EBRT but can be difficult to interpret due to treatment related changes in the prostate and spatial resolution [6–8]. Therefore, to confirm recurrence or residual disease, biopsy is usually performed.

Biopsy has also been investigated as a stand-alone technique to evaluate local PCa recurrence after EBRT, albeit much less frequently [9]. Post-EBRT biopsies taken <2 years after EBRT treatment are not reliable and can still be challenging to interpret, for instance when there is cancer and treatment effect leading to ‘indeterminate’ reports alongside distinctly positive and negative [10]. Nevertheless, positive biopsy after EBRT implies failure of local tumour control and is associated with a downward trend in PCa prognosis [11], and in certain cases a positive biopsy can be found before BCF appears [12]. While a post-EBRT positive biopsy cannot rule out metastasis, it does provide direct histological evidence of local disease which could be targeted with salvage therapies.

A better understanding of the positive biopsy rate after EBRT also offers an opportunity to compare EBRT to other treatment modalities. To properly assess oncological efficacy, it is often expected that any new curative PCa treatment performs a post-treatment biopsy to verify the absence of cancer. Understanding the positive biopsy rate after EBRT will provide a valuable measuring stick.

In this meta-analysis we systematically reviewed the relevant literature and determined the post-EBRT positive biopsy rate when the biopsy was performed at least 2 years after treatment. We also assessed the associated risk of a of a positive biopsy vs. a negative biopsy as an indicator for long-term PCa outcome.

## Methods

### Literature search and study selection

We searched for studies that utilised EBRT alone or in combination with androgen deprivation therapy (ADT) as primary treatment for low to intermediate risk PCa (PSA ≤ 20 ng/ml, Gleason score ≤ 7, clinical stage ≤ T2b), where a biopsy at ≥2 years post-EBRT was an endpoint or observation of the study. Studies with high-risk PCa were accepted if they also included low- or intermediate-risk PCa in their patient population. Brachytherapy which is often combined with EBRT and proton beam therapy were not included, in order to focus on EBRT. Study eligibility criteria are summarised in Table S1.

Preferred Reporting Items for Systematic Reviews and Meta-Analyses (PRISMA) guidelines were utilised to search PUBMED/MEDLINE and EMBASE. We defined study eligibility with reference to Population, Intervention, Outcome, and Study design. A structured literature search for studies until March 2020 with keywords “prostatic neoplasms”; “prostate cancer”; “biopsy”; “radiotherapy”; and not “brachytherapy” was conducted (Supplementary material Table S2). The separate database searches were imported into Mendeley Desktop (Mendeley, London, United Kingdom) to detect and remove duplicates. Search results containing editorials, review, case-reports, models and opinion-pieces were automatically removed from consideration. Thereafter, the remaining search results were screened by two authors (SS and SB) for relevant keywords in the title and abstract. Only randomised controlled trials or cohort studies published in English journals were used. In cases where two or more studies reported results of an overlapping patient cohort, the one with higher number of patients biopsied at ≥2 years was selected. In one case the overlapping patient cohort (Zelefsky et al. [12] and Zelefsky et al. [13]) was clarified by contacting the study author directly. A detailed breakdown of the rejected studies is described in the Supplementary Material (Table S3).

### Data extraction

A variety of information was extracted by the same two authors (SS and SB) from each eligible study, including: PCa risk group breakdown, EBRT technique, dose, number of patients who received a biopsy ≥2 years after EBRT, and the positive/indeterminate biopsy rate. The baseline PCa risk stratification was not consistent across studies, including the clinical T stage, Gleason score and National Comprehensive Cancer Network (NCCN) risk assessments. If available, the 5–10-year long-term data on PCa progression such as biochemical failure (BCF), distant metastases-free survival (DMFS) and prostate cancer-specific mortality (PCSM) was also recorded. If high-risk PCa was also included in the study population, the number of patients counted, the post-EBRT positive biopsy rate and any long-term outcomes were recorded for only the low- to intermediate-risk group when possible, although some studies did not differentiate between risk groups.

### Assessment of study quality

A validated quality assessment tool was used to evaluate the quality of the studies that met our eligibility criteria by two authors (SS and SB) [14, 15]. This tool utilising a series of questions applied to each individual study to address study objectives, study population, the intervention and any co-interventions, the outcome measures, statistical analysis, results and conclusion and disclosures.

### 2020 NCCN Guidelines

The NCCN has written guidelines on recommended EBRT dose and fractionation based on existing clinical evidence [16], summarised in Table S4 for very low to unfavourable intermediate risk PCa. A subgroup analysis of those studies that used dosing regimens consistent with the 2020 NCCN guidelines for EBRT (including hypofractionated regimens) was performed.

### Statistical analysis

Data collection and basic analysis was performed in Excel (Microsoft Corporation, Redmond, CA, USA). To determine a specific positive and indeterminate biopsy rate across a selection of studies, a weighted average of each study was performed, which combines both the reported biopsy rate and the number of patients treated:

$${\mathrm{Individual}}\,{\mathrm{weights}}\,(W_i) = N_{{\mathrm{biopsied}}}\frac{{{\mathrm{\% }}{\mathrm{biopsy}}\,{\mathrm{positive}}}}{{N_{{\mathrm{total}}}}}$$

$${\mathrm{Weighted}}\,{\mathrm{average}} = \mathop {\sum }\limits_{i = 0}^n W_i$$

95% confidence intervals (CI) for the positive and indeterminate biopsy rates were calculated by obtaining the total number of patients that underwent a biopsy and the number of positive/indeterminate biopsies from each individual study. This data was entered into MedCalc (Medcalc Software Ltd, Ostend, Belgium) and using a Freeman-Tukey transformation of proportions under assumption of random effects an overall CI was derived. This technique was applied for all subgroup analysis.

For those studies where sufficient information was provided, the odds ratio (OR) of biopsy outcome on biochemical failure (BCF), distant metastases-free survival (DMFS) and prostate cancer-specific mortality (PCSM) was calculated, in a manner similar to [17]. Briefly, studies were only included if they reported both the number of positive/negative biopsies as well as the corresponding outcome between a specific positive/negative biopsy outcome and the presence/absence of BCF, DMFS and PCSM. Patients who did not undergo a biopsy, or whose biopsy finding was indeterminate, were not included in the analysis. The time to failure was not accounted for in the model.

For each eligible study a 2 × 2 contingency table was be computed (Table S5). We calculated the total number of patients who had a positive or negative biopsy, and the proportion from each group that had BCF, DMFS and PCSM. The data was input into Cochrane Review Manager Software v5.3 (Cochrane, London, United Kingdom) and this software was used to statistically pool odds ratios (OR), CI, *p*-values and additionally characterise data heterogeneity. The dichotomous Mantel–Haenszel technique with a random effects model and confidence intervals of 95% were used.

## Results

### Search results - overall

**Fig. 1:**
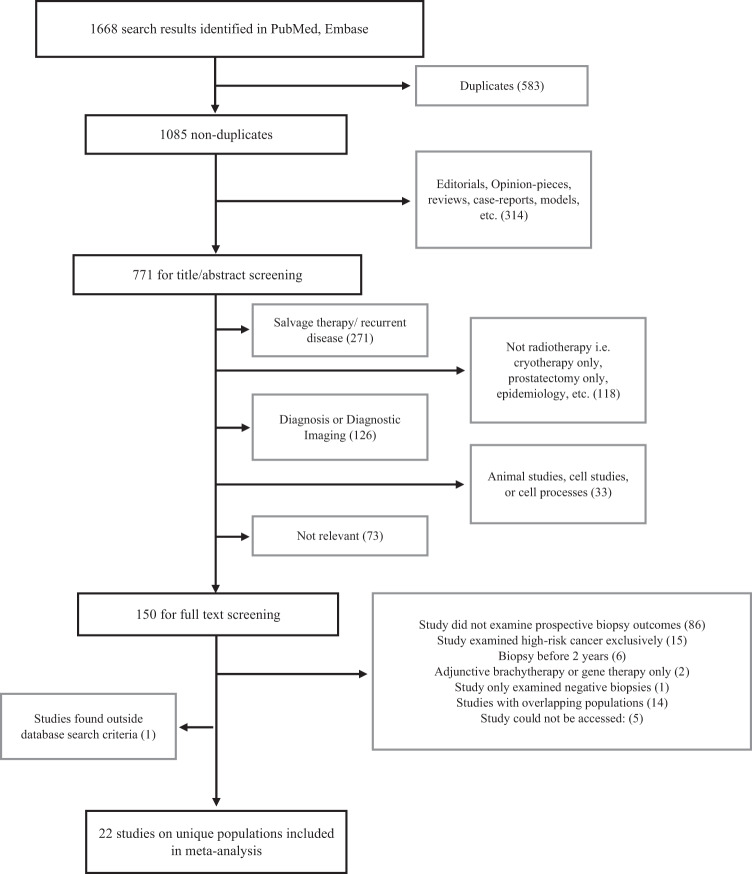
Study selection. Flow diagram summarising selection of studies that meet inclusion criteria.

The selection process is shown in Fig. 1. Twenty-two studies satisfied the eligibility criteria, including 10 randomised controlled trials and 12 cohort studies (Table 1).

**Table 1:** Studies meeting eligibility criteria.

Article number	Author	Pub. year	Patient Risk Group	Technique	Total dose	Fraction	EQD2, α/β = 2.5	Number patients in entire study with low- or intermediate-risk Pca	Number biopsied at ≥24 months	Biopsy Study protocol	Consistent with 2020 NCCN guidelines
1	Kiesling et al. [23]	1980	n/a	4-field box	60-70	n/a	n/a	68	24	Not reported	No^b^
2	Forman et al. [24]	1993	86.7% Low to Int 13.3% High	3D-CRT	66.5 (65–69)	1.8–2	62–69	30	30	Part of Study protocol	No
3	Ljung et al. [25]	1995	76.4% Low to Int 23.6% High	4-field box	71.7 (60–74)	2–2.3	60–75	70	55	Encouraged to undergo post-treatment biopsy	No^b^
4	Laverdière et al. [26]	1997	73.9% Low to Int 26.1% High	4-field box	64	2	64	120	68	Part of Study protocol	No^b^
5	Almroth et al. [27]	1998	37.5% Low to Int 67.5% High	4-field box	68.1 (64.5–72.5)	1.8	61–69	36	24	Encouraged to undergo post-treatment biopsy	No
6	Crook et al. [11]	2000	88% Low to Int 12% High	4-field box, 3D-CRT	66	1.8–2	63–66	498	156	Part of Study protocol	No^b^
7	Pollack et al. [19]	2002	78.5% Low to Int 22.5% High	4-field box, 3D-CRT	70–78	2	70–78	201	158	Part of Study protocol	Yes^a^^b^
8	Nichol et al. [28]	2005	89% Low to Int 11% High	3D-CRT	75.6 (65–76)	1.8	62–72	140	71	Part of Study protocol	Yes^b^
9	Lukka et al. [22]	2005	90.7% Low to Int 9.3% High	4-field box	52.5–66	2–2.6	60–66	936	682	Part of Study protocol	No^b^
10	Martin et al. [18]	2007	92.4% Low to Int 7.6% High	Image-guided IMRT	60	3	73	92	25	Encouraged to undergo post-treatment biopsy	Yes^b^
11	Zelefsky et al. [29]	2008	58% Low to Int 42% High	3D-CRT, IMRT	75.6–81	1.8	67–77	1773	339	Encouraged to undergo post-treatment biopsy	Yes^a^
12	Donnelly et al. [30]	2010	31.1% Low to Int 68.9% High	4-field box	68–73.5	2	68–74	122	76	Part of Study protocol	No^b^
13	Solberg et al. [31]	2011	12.5% Low to Int 87.5% High	3D-CRT	70	2	70	120	55	Part of Study protocol	No^b^
14	Loblaw et al. [32]	2013	100% Low to Int	SBRT	35	7	74	84	71	Part of Study protocol	No
15	Petrongari et al. [33]	2013	100% Low to Int	IMRT Dose Escalation	86	2	86	39	17	Part of Study protocol	No
16	Freytag et al. [34]	2014	100% Low to Int	IMRT	80	2	80	23	19	Part of Study protocol	Yes
17	Krauss et al. [20]	2015	100% Low to Int	Varied based on site	66.6	1.8	64	1755	831	Part of Study protocol	No
18	Huang et al. [35]	2015	87.2% Low to Int 12.8% High	CIMRT/HIMRT	70.2–76	2–2.7	76–81	303	86	Part of Study protocol	Yes^b^
19	Kass-Iliyya et al. [21]	2018	85.3% Low to Int 14.7% High	Varied based on site	74	2	74	843	312	Part of Study protocol	Yes^a^
20	Zelefsky et al. [12]	2018	100% Low to Int	SBRT	32.5–35	6.5–7	65–74	136	47	Encouraged to undergo post-treatment biopsy	No
21	Zelefsky et al. [13]	2019	100% Low to Int	SBRT	37.5–40	7.5–8	83–93	551	119	Encouraged to undergo post-treatment biopsy	Yes
22	Zapatero et al. [36]	2019	25.4% Low to Int 75.4% High	3D-CRT	77 (66–84)	1.8–2	76–80	232	232	Part of Study protocol	Yes^a^

*3D-CRT* three-dimensional conformal radiotherapy, *IMRT* image-guided radiotherapy, *CIMRT* conventional IMRT, *HIMRT* hypofractionated IMRT, *SBRT* stereotactic body radiotherapy, EQD2 equivalent dose in 2 Gy fractions, NCCN National Comprehensive Cancer Network.

^a^Only a percentage of all treated patients received their EBRT treatment according to 2020 NCCN guidelines.

^b^Low- and intermediate-risk patients could not be separated from high-risk.

### Search results – post-EBRT biopsy protocol

Differences were observed in the post-EBRT biopsy protocol, which is summarised in Table 1. Post-EBRT biopsy was mandated in 68% of all studies (15/22), while patients were encouraged to undergo a post-EBRT biopsy in 27% of studies (6/22). One study did not disclose their post-EBRT biopsy methodology. Even if the post-EBRT was mandated at enrolment, many patients did not undergo their follow-up biopsy. As a result, the biopsy follow-up rates varied considerably ranging from 19% to 100% (median 59%).

### Search results – PCa risk group

Seven of twenty-two (7/22) studies included exclusively low- or intermediate-risk patients, with the remaining 15/22 studies including some population of high-risk patients. Of these 15 studies including any population of high-risk patients, only four studied predominantly high-risk patients, which accounts for 10% of all patients included in the entire meta-analysis, and therefore the remaining 90% of patients in the meta-analysis were either completely or predominantly low- to intermediate-risk. The risk dependent post-EBRT positive biopsy rate was directly extracted in exactly half of the 22 studies, while the combined positive biopsy rate across all risk groups was used for the remainder.

### Study quality evaluation

The results of the study quality assessment are described in Table 2. Most studies met the statements of the quality assessment tool (mean 81%, range 41–100%). Some older studies did not mention competing interests or funding support (41%). Nine studies (41%) did not report adverse events because they focused on oncological outcomes similar to this review. Overall, according to quality assessment criteria, the quality of the studies included was high.

**Table 2:** Modified Delphi technique used to assess study quality on the 22 studies which met eligibility criteria.

Criteria	Studies, *n* (%)	
	Yes	No
*Study objective*		
1. Is the hypothesis/aim/objective of the study clearly stated in the abstract, introduction, or methods section?	22 (100)	0 (0.0)
*Study population*		
2. Are the characteristics of the participants included in the study described?	21 (95)	1 (5.0)
3. Were the cases collected in more than 1 Centre?	9 (41)	13 (59)
4. Are the eligibility criteria to enter the study explicit and appropriate?	15 (68)	7 (32)
5. Did participants enter the study at a similar point in the disease?	21 (95)	1 (5.0)
*Intervention and co-intervention*		
6. Was the intervention clearly described in the study?	21 (95)	1 (5.0)
7. Were additional interventions (co-interventions) clearly reported in the study?	20 (91)	2 (9.0)
*Outcome measures*		
8. Are the outcome measures clearly defined in the introduction or methods section?	22 (100)	0 (0.0)
9. Were relevant outcomes appropriately measured with objective/or subjective methods?	22 (100)	0 (0.0)
10. Were outcomes measured before and after the intervention?	19 (86)	3 (14)
*Statistical analysis*		
11. Were the statistical tests used to assess the relevant outcomes appropriate?	16 (73)	6 (27)
*Results and conclusions*		
12. Was the length of follow-up reported?	22 (100)	0 (0.0)
13. Was the loss of follow-up reported?	21 (95)	1 (5.0)
14. Does the study provide estimates of the random variability in the data analysis of relevant outcomes?	16 (73)	6 (27)
15. Are the adverse events reported?	9 (41)	13 (59)
16. Are the conclusions of the study supported by the results?	17 (77)	5 (23)
*Competing interest and source of support*		
17. Are both competing interest and source of support for the study reported? [37–39]	9 (41)	13 (59)

### Overall biopsy rates

The weighted-average positive biopsy rate across the 22 studies was 32% (95% CI: 25–39%, range: 4.0–67%), which includes a total of 3017 patients biopsied at ≥2 years. This positive biopsy rate does not discriminate based on the trial design, how EBRT was delivered, whether ADT was administered, whether the radiation dose conformed to the 2020 NCCN guidelines, or how the post-EBRT biopsy protocol was defined. Across nine studies that reported it, indeterminate biopsy was identified in a weighted-average of 22% (95% CI: 14–28%, range: 5.9–39%) of patients.

### Sensitivity analysis

Several differences across the available studies were observed. A sensitivity analysis of potentially impactful variables was performed (Table 3), along with the respective 95% CI.

**Table 3:** Factors that influence post-EBRT positive biopsy rate.

	All studies	Consistent with 2020 NCCN guidelines	Consistent with 2020 NCCN guidelines, no ADT	Consistent with 2020 NCCN guidelines + short-term ADT	Mandated biopsy	Mandated biopsy and managed to biopsy ≥70% of all patients	Exclusively low- to intermediate-risk patients	Exclusively high-risk patients
No. of studies	22	9	5	3	15	4	11	5
No. of patients	3067	832	349	241	2450	798	1567	357
Positive biopsy rate (95% CI)	32% (25–39)	22% (19–41)	34% (23–50)	14% (3.8–31)	35% (21–38)	47% (5–63)	25% (15–32)	29% (20–46)

### Positive biopsy rate vs. NCCN 2020 guidelines, short-term ADT vs. no ADT

Nine studies used dosage regimens consistent with the current 2020 NCCN guidelines directly or had populations of patients that did (Table 3). It was possible in 8/9 studies to extract the positive biopsy rate for those patients who received dose rates that were consistent with the NCCN 2020 guidelines. One study (Nichol et al.) did not specify the positive biopsy rate as a function of dose rate. However, over 90% of patients in this specific study received a dose regimen consistent with NCCN 2020 guidelines, which was deemed acceptable. This led to a total of 832 patients with a weighted-average positive biopsy rate of 22% (95% CI: 19–41%, range: 3.6–58%).

Within the same subgroup, five of nine studies reported their post-EBRT positive biopsy rate of 34% (95% CI: 23–50%, range: 12–58%) across 349 patients without any ADT usage. On the other hand, it was found that the weighted positive biopsy rate in combination with short-term (3–6 months) ADT was 14% (95% CI: 3.8–31%, range: 3.6–32%), across 241 patients. No information on long-term ADT was clearly reported.

### Positive biopsy rate vs. follow-up biopsy protocol

Fifteen of twenty-two studies mandated a post-EBRT biopsy in their protocol, resulting in a weighted positive biopsy rate of 35% (95% CI: 21–38%, range: 4.2–58%) across 2450 patients. Within this 15-study subgroup, only 4/15 studies prospectively biopsied ≥70% of patients, and a 47% (95% CI: 5–63%, range: 4.2–58%) weighted positive biopsy rate across 798 patients was observed (Forman et al., Lukka et al., Loblaw et al. and Freytag et al.). The remaining 11/15 studies had a weighted positive biopsy rate of 30% across 2279 patients (95% CI: 22–37%, range: 5.9–44%). The subgroup of six studies which did not mandate but merely encouraged patients to undergo a post-EBRT biopsy had a weighted positive biopsy rate of 29% (95% CI: 21–52%, range: 12–67%) across 438 patients.

### Positive biopsy rate vs. baseline PCa risk-group

The risk-group dependence on positive biopsy rate was extracted in 11/22 studies for low- and intermediate-risk disease combined and 5/22 studies for high-risk disease only. Across a pool of 1567 patients, the weighted positive biopsy rate after EBRT was 25% (95% CI: 15–32%, range: 4.2–67%) for low- and intermediate-risk disease combined, which increased to 29% (95% CI: 20–46%, range: 12–67%). across a pool of 357 patients for high-risk disease.

### Risk associated with positive biopsy and BCF, DMFS and PCSM

**Fig. 2:**
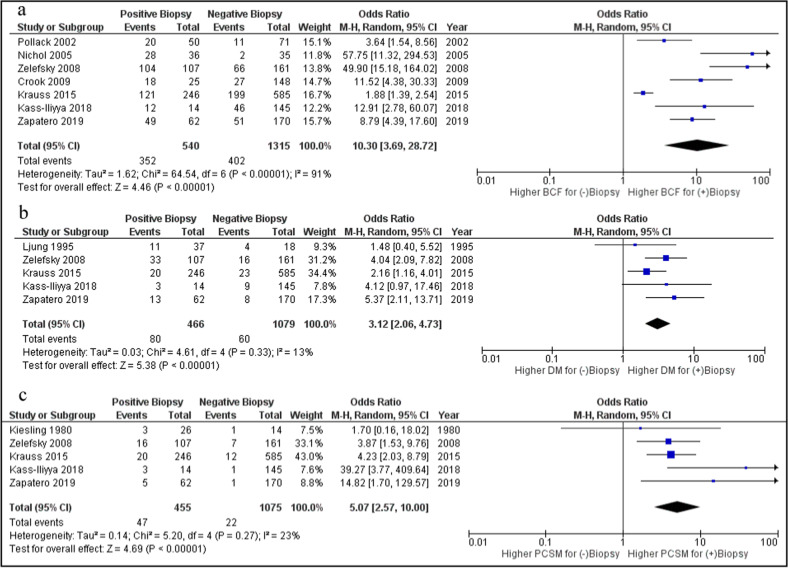
Odds ratio for patients with positive post-EBRT positive biopsy at ≥2 years follow-up. **a** Risk of biochemical failure (BCF), **b** risk of distant metastasis (DM), and **c** risk of prostate cancer-specific mortality (PCSM).

In this study we also assessed the relationship between positive biopsy at ≥2 years after EBRT with oncologically relevant longer-term outcomes of BCF, DMFS, and PCSM. From a pool of 1855 patients, those with a positive post-EBRT biopsy had ~10-fold higher odds of developing BCF than those with negative biopsy (OR 10.3, 95% CI: 3.7–28.7, *p* < 0.00001), with a weighted absolute BCF rate of 67% vs. 29% for positive and negative biopsy, respectively (Fig. 2a). From a pool of 1545 patients, those with a positive-EBRT biopsy had approximately three times higher odds of developing distant metastasis than those with negative biopsy (OR 3.1, 95% CI: 2.1–4.7, *p* < 0.00001), with a weighted absolute distant metastases rate of 17% vs. 5.6% for positive and negative biopsy, respectively (Fig. 2b). Lastly, from a pool of 1530 patients, those with a positive-EBRT biopsy had five times higher odds of dying from their PCa than those with negative biopsy (OR 5.1, 95% CI: 2.6–10, *p* < 0.00001), with a weighted absolute PCSM rate of 10% vs. 2.1% for positive and negative biopsy, respectively (Fig. 2c). It should be noted that there was heterogeneity observed across noted regarding the relationship between a positive biopsy and BCF, with an *I*^2^ statistic of 91% from Fig. 2a, and is likely a consequence of the variable positive biopsy rate observed in the studies. There was much lower heterogeneity for DM and PCSM, *I*^2^ statistic 13% and 23% respectively.

## Discussion

This meta-analysis included 22 studies which reported biopsy at ≥2 years post-EBRT as an endpoint or study observation and utilised EBRT alone or in combination with androgen deprivation therapy (ADT) as primary treatment for low to intermediate risk PCa (PSA ≤ 20 ng/ml, Gleason score  ≤ 7, clinical stage ≤ T2b). The overall post-EBRT positive biopsy rate at least 2 years after treatment was 32%, although there was a variable biopsy rate in these studies which introduces bias. A subgroup analysis was performed to include only studies that prospectively mandated biopsy in their study protocol, resulting in a positive biopsy rate of 35% from 15 studies. Out of these studies, perhaps the most relevant results for understanding the rate of persistent local disease 2 years after EBRT, come from four studies that had high compliance to prospectively mandated biopsy (biopsy of ≥70% of all patients) resulting in a positive biopsy rate of 47%.

Other subgroup analyses were performed to account for modern dose regimens consistent with NCCN guidelines (positive biopsy rate 22%), baseline PCa risk group (positive biopsy rate 25% for low- to intermediate-risk only) and the addition/removal of ADT (positive biopsy rate 14% vs. 34%, ADT vs. no ADT). These findings illustrate the positive impact of modern dosing regimens, ADT and patient risk group on local disease control after EBRT.

In our analysis, 5–10-year follow-up data showed a positive biopsy post-EBRT was associated with higher odds of BCF, DMFS and PCSM by 10.3, 3.1 and 5.1 times, respectively. These associated poor outcomes due to failure of local tumour control are likely to represent patients with ‘radio-resistant’ tumours. These tumours may have a different tumour biology to radio-sensitive tumours resulting in higher local recurrence and poorer outcomes. Further research is needed to characterise the tumour biology in these patients and find biomarkers which could help identify these patients early to prevent poor outcomes after EBRT.

Salvage therapies have evolved for patients with local recurrence after EBRT. Non-surgical options such as high intensity focused ultrasound (HIFU), cryotherapy and brachytherapy are now available for patients who may not be fit for salvage prostatectomy. Although no randomised controlled trial exists to compare all the different modalities, several case series suggest comparable oncological outcomes [15, 37–39]. The findings of this study suggest that patients should be counselled about potential poor oncological outcomes if they have a local recurrence and adds weight to the need for active treatment of radiorecurrent prostate cancer.

There are several limitations in this meta-analysis. Perhaps most importantly, the cohort biopsy rate in the included studies was variable and not mandated in a few studies. This is a source of considerable bias in studies reporting positive biopsy rates with low cohort biopsy rates and consequently in this meta-analysis. In those studies where a biopsy was not mandatory, it is conceivable that patients with suspicious biochemical measurements were nevertheless more likely to receive one [18], which may have inflated the aggregate positive biopsy rate. Conversely, there are possible influencers which may have had the opposite effect. For instance, in certain trials patients who experienced BCF prior to the 2-year biopsy follow-up were removed from the study and not counted, likely lowering the reported positive biopsy rate [10, 19–21]. Therefore, to discern an unbiased true positive biopsy rate, more studies are needed that mandate biopsy in their protocols and biopsy a high percentage of patients. It is clear from this study that even when biopsy is mandated prospectively in study protocols, the cohort biopsy rate is variable, likely because of its invasive nature. Although this introduces considerable bias to the analysis, it may reflect ‘real world’ clinical practice, where patients often refuse post-treatment biopsy. Post treatment biopsy still remains contested as a measure of post-radiotherapy outcome due to its limitations of under-sampling, delayed histological resolution, and equivocal post-treatment histology [10, 21, 22]. Though these limitations were accounted for in the sensitivity analysis, their potential effect on positive biopsy rate cannot be discounted. Furthermore, prostate biopsy does not capture recurrences outside the gland. Since the number of studies reporting long-term oncological outcomes data was small, and there was heterogeneity between EBRT technique especially in older studies and study populations, this analysis is subject to publication and reporting bias.

In conclusion, this meta-analysis shows comparable positive biopsy rates at two years post EBRT treatment compared with other treatment modalities. A positive biopsy after radiotherapy compared to a negative biopsy has higher odds of poor long-term outcome.

## Supplementary information

Supplemental Material

Supplementary table S1

Supplementary table S2

Supplementary table S3

Supplementary table S4

Supplementary table S5
