# Supplementary material for: Long-term biopsy outcomes in prostate cancer patients treated with external beam radiotherapy: a systematic review and meta-analysis
Source: Prostate Cancer Prostatic Dis. 2021 Feb 8;24(3):612–22. doi: 10.1038/s41391-021-00323-6 (PMC8384630; doi:10.1038/s41391-021-00323-6)
Supplement: Supplementary file 1 — Supplemental Material [file 41391_2021_323_MOESM1_ESM.docx]

**Supplemental Material**

**Table S1** – Study eligibility

**Table S2** – Search terms

**Table S3** – Exclusions based on search results

**Table S4** - 2020 National Comprehensive Cancer Network (NCCN) guidelines used to define the subset of studies employing modern external-beam radiotherapy (EBRT) dose and fractionation protocols.

**Table S5 –** 2X2 Contingency Table
