# Supplementary material for: Long-term biopsy outcomes in prostate cancer patients treated with external beam radiotherapy: a systematic review and meta-analysis
Source: Prostate Cancer Prostatic Dis. 2021 Feb 8;24(3):612–22. doi: 10.1038/s41391-021-00323-6 (PMC8384630; doi:10.1038/s41391-021-00323-6)
Supplement: Supplementary file 3 — Supplementary table S2 [file 41391_2021_323_MOESM3_ESM.docx]

| **Table S2. Search terms** | |
| --- | --- |
| **Database** | **Search Terms** |
| PubMed  1069 results | (("prostatic neoplasms"[MeSH Major Topic] AND biopsy[Text Word]) AND "radiotherapy"[Text Word]) NOT "brachytherapy"[MeSH Terms] AND (Journal Article[ptyp] AND English[lang]) |
| Ovid (MEDLINE + Embase)  599 results | 1. (prostatic neoplasms.sh. and biopsy.tw. and radiotherapy.tw.) not brachytherapy.sh. 2. Limit (1) to English Language 3. Limit (2) to human |
| Manual Search in PUBMED, 1 result | Keywords *prostate cancer, biopsy, radiotherapy* and NOT *brachytherapy* |
