# Supplementary material for: Long-term biopsy outcomes in prostate cancer patients treated with external beam radiotherapy: a systematic review and meta-analysis
Source: Prostate Cancer Prostatic Dis. 2021 Feb 8;24(3):612–22. doi: 10.1038/s41391-021-00323-6 (PMC8384630; doi:10.1038/s41391-021-00323-6)
Supplement: Supplementary file 4 — Supplementary table S3 [file 41391_2021_323_MOESM4_ESM.docx]

| **Table S3. Exclusions based on search results** | |
| --- | --- |
| **Studies removed for being not relevant** is a catch-all category for studies that may have used radiotherapy, but did not satisfy specific eligibility criteria**:** | - Pre-treatment biopsy only: 37 - Focus on metastases/seminal vesicles: 17 - Focus on adverse events only: 6 - Focused on complementary therapies: 3 - Rhabdomyosarcoma: 2 - Focus on biochemical failure only: 6 - Pharmaceuticals: 2 |
| **Studies with** **overlapping populations**  Studies were suspected of overlap if patient enrolment years and treatment location coincided with one another. The study with more patients enrolled were used. | - Kiesling, Nachtsheim: 2 studies (1 overlapping removed) - Zelefsky, Levegrun: 6 studies (5 overlapping removed) - Crook, Nichol, Martin: 7 studies (4 overlapping removed) - Freytag 2 studies (1 overlapping removed) Pollack, Vance: 2 studies (1 overlapping removed) - Jones, Krauss 2 studies (1 overlapping removed) - Zapatero 2 studies (1 overlapping removed) |
| ****** Studies that could not be accessed** | Hadley, D. A., Herr, H. W., & Wuerker, R. B. (1979). Evaluation of postirradiated prostate biopsy for prostatic carcinoma. *Surgical Forum*, *30*, 572–574.  Leach, G. E., Cooper, J. F., Kagan, A. R., Snyder, R., & Forsythe, A. (1982). Radiotherapy for prostatic carcinoma: post-irradiation prostatic biopsy and recurrence patterns with long-term followup. *The Journal of Urology*, *128*(3), 505–509.  Kurup, P., Kramer, T. S., Lee, M. S., & Phillips, R. (1984). External beam irradiation of prostate cancer. Experience in 163 patients. *Cancer*, *53*(1), 37–43.  Bagshaw, M. A., Ray, G. R., & Cox, R. S. (1985). Radiotherapy of prostatic carcinoma: long- or short-term efficacy (Stanford University experience). *Urology*, *25*(2 Suppl), 17–23.  Gabriele, D., Garibaldi, M., Girelli, G., Taraglio, S., Duregon, E., Gabriele, P., … Bollito, E. (2016). Percentage of positive prostate biopsies independently predicts biochemical outcome following radiation therapy for prostate cancer. *Panminerva Medica*, *58*(2), 109–114. |
